# Modulation of Radiation Response by the Tetrahydrobiopterin Pathway

**DOI:** 10.3390/antiox4010068

**Published:** 2015-01-22

**Authors:** Rupak Pathak, Amrita K. Cheema, Simina M. Boca, Kimberly J. Krager, Martin Hauer-Jensen, Nukhet Aykin-Burns

**Affiliations:** 1 Division of Radiation Health, Department of Pharmaceutical Sciences, College of Pharmacy, University of Arkansas for Medical Sciences, Biomed II, Room 441A-2, 4301 West Markham #522-10, Little Rock, AR 72205, USA; E-Mails: RPathak@uams.edu (R.P.); KJKrager@uams.edu (K.J.K.); MHJensen@uams.edu (M.H.-J.); 2 Department of Oncology, Georgetown University Medical Center, Washington, DC 20057, USA; E-Mails: Amrita.Cheema@georgetown.edu (A.K.C.); smb310@georgetown.edu (S.M.B.); 3 Department of Biochemistry, Molecular and Cellular Biology, Georgetown University Medical Center, Washington, DC 20057, USA; 4 Innovation Center for Biomedical Informatics, Georgetown University Medical Center, Washington, DC 20057, USA; 5 Surgical Service, Central Arkansas Veterans Healthcare System, Little Rock, AR 72205, USA

**Keywords:** ionizing radiation, metabolomics, oxidative stress, tetrahydrobiopterin

## Abstract

Ionizing radiation (IR) is an integral component of our lives due to highly prevalent sources such as medical, environmental, and/or accidental. Thus, understanding of the mechanisms by which radiation toxicity develops is crucial to address acute and chronic health problems that occur following IR exposure. Immediate formation of IR-induced free radicals as well as their persistent effects on metabolism through subsequent alterations in redox mediated inter- and intracellular processes are globally accepted as significant contributors to early and late effects of IR exposure. This includes but is not limited to cytotoxicity, genomic instability, fibrosis and inflammation. Damage to the critical biomolecules leading to detrimental long-term alterations in metabolic redox homeostasis following IR exposure has been the focus of various independent investigations over last several decades. The growth of the “omics” technologies during the past decade has enabled integration of “data from traditional radiobiology research”, with data from metabolomics studies. This review will focus on the role of tetrahydrobiopterin (BH4), an understudied redox-sensitive metabolite, plays in the pathogenesis of post-irradiation normal tissue injury as well as how the metabolomic readout of BH4 metabolism fits in the overall picture of disrupted oxidative metabolism following IR exposure.

## 1. Introduction

Both targeted radiotherapy to treat malignancies and nuclear/accidental irradiation exposure are known to cause injury to normal tissues, limiting the therapeutic dose range in clinical settings or causing mass casualties [1,2,3]. Therefore, there is an imminent and unmet need to develop radiation mitigators and improve overall quality of life of individuals who are exposed to irradiation by any means. There is emerging awareness of the concept that total body radiation injury is due to multi organ dysfunction syndromes [4,5,6,7,8]. To foster feasible strategies to mitigate radiation induced multi organ tissue damage, it is imperative to identify the biological targets of Ionizing radiation (IR) and understand the interactions of these targets with each other within the entire biochemical and molecular machinery of human physiology.

A metabolomics approach offers to detect and quantitate changes in the levels of metabolites that directly reflect the overall physiological status of an individual [9]. It provides a systems wide biological snapshot of the net expression of metabolites from several known pathways in response to exogenous challenges such as IR. Cellular radiation response and damage is a result of both direct and indirect effects of radiation. Direct radiation damage is caused by specific bond breakage or modification within biomolecules including DNA, proteins and lipids. While radiolysis of water leads to the rapid generation of reactive oxygen species (ROS) resulting in oxidative stress [10,11], which is characterized by dramatic effects on cellular function; including gene expression, transcription factor activation, redox sensitive signaling pathways. All of these effects contribute to IR-induced signaling and adaptive responses, which feed into endogenous metabolism. Therefore, utilizing a metabolomics approach to study IR-induced normal tissue injury would provide invaluable information complementary to traditional radiobiology as well as genomics and proteomics studies.

Recent advances in high-resolution chromatography and mass spectrometry have enabled researchers to use this approach to discover and develop predictive biomarkers for radiation injury [12,13,14,15,16]. IR dependent metabolomics changes have already been reported in human cell lines and mouse urine, indicating multiple oxidation sensitive targets in their metabolic profiles shortly following exposure [17,18,19].

## 2. Cellular Redox Responses Following Radiation Exposure

The generation of ROS and reactive nitrogen species (RNS) following IR exposure, such as superoxide and hydrogen peroxide (H_2_O_2_), and peroxynitrite anion and peroxynitrous acid, respectively, cause further damage to DNA and cellular compartments. The damage leads to an imbalance in the stoichiometry of biochemical reactions and perturbation of critical pathways [20,21,22,23,24,25,26,27,28]. This deviation from cellular homeostasis then leads to a cascade of events involving additional formation of intracellular oxidants and reductants via the mitochondrial electron transport chain, stress mechanisms, and altered expression of ROS producing enzymes. Thus, it is becoming more evident that disruption in intracellular metabolic redox homeostasis will remain compromised for longer periods of time following IR [11,29,30].

While a great deal is known about the initial ROS formation shortly after IR exposures, the contribution of the effected enzymatic and/or non-enzymatic antioxidant machinery due to the formation of RNS is only beginning to be established. Although a cell is equipped with a variety of antioxidants to detoxify increased levels of ROS and RNS, they may rapidly become depleted following radiation exposure.

Tetrahydrobiopterin (BH4) is one of the critical cellular non-enzymatic redox sensitive antioxidants. It plays several critical roles in diverse bio-chemical pathways because of its cofactor function for a number of enzymes, such as aromatic amino acid hydroxylases and nitric oxide synthases (NOSs) [31]. Oxidation of BH4 to dihydrobiopterin (BH2) and other oxidized biopterin species causes endothelial NOS (eNOS) to produce higher superoxide levels instead of nitric oxide (NO), a phenomenon popularly termed as “eNOS uncoupling” resulting in increased oxidative stress [32]. The oxidation of BH4 to BH2 also further uncouples eNOS due to the ability of BH2 to compete and displace BH4 from eNOS. BH4 insufficiency-dependent eNOS uncoupling has been suggested as an etiologic factor in the progression and the subsequent development of various neurological, cardiovascular and fibrotic diseases [33]. BH4 treatment suppresses these oxidative stress-dependent adverse pathophysiological conditions [34]. Therefore, maintenance of cellular BH4 homeostasis is critical for normal physiological functioning. This critical balance of BH4 can be altered by differential intrinsic as well as extrinsic stimuli, including IR.

## 3. Radiation Effect on BH4 and Peroxynitrite Formation

Although literature describing the effect of IR on BH4 is limited, recent* in vivo* studies have shown that IR causes decreased BH4 level in tissue [35]. Berbee* et al.*, (2010) showed that total body irradiation (TBI) of mice with 8.5 Gy of γ-ray suppressed BH4 bioavailability in lung tissue samples at 3.5 day [36]. However, the authors observed the initial decline returned to basal levels at day 7 and a compensatory increase in lung BH4 levels at later post-irradiation time points (day 14 and day 21) [36]. Similarly, a significant reduction in the BH4 level as well as BH4/BH2 ratio, which is considered as a critical determinant of eNOS uncoupling, was noticed in the lung tissue of mice 24 h following 8.5 Gy of TBI [35]. This insufficiency of BH4 availability leads to eNOS uncoupling-mediated higher superoxide generation, which in turn may react with NO to produce peroxynitrite; a powerful toxic oxidant that enhances nitrosative stress in biological system. Peroxynitrite can also oxidize BH4 to BH2 forming a vicious cycle of eNOS uncoupling [37]. The rate constant for peroxynitrite and BH4 reaction is 6–10 times greater than peroxynitrite and many cellular antioxidants making BH4 a major oxidative target [38]. Deficiency in NO or enhanced production of peroxynitrite is a critical marker of endothelial dysfunction and a major cause of pathogenesis of fibrotic diseases [39]. Berbee* et al.*, (2010) reported that IR, in addition to BH4 deficiency, also causes increased production of peroxynitrite in mice aorta after exposure to 8.5 Gy of TBI [36], indicating a function mediated by uncoupled eNOS as a result of IR-induced BH4 unavailability. To further determine whether IR-induced aortal peroxynitrite formation is BH4-eNOS-dependent, they treated irradiated mice with BH4 or NH4 (a compound with similar antioxidant property like BH4, but with no eNOS co-factor function) and measured aortal peroxinitrite formation at different post-irradiation time points. Interestingly, they found BH4, but not NH4, was able to suppress IR-induced peroxynitrite formation, which clearly indicates BH4-dependent eNOS function is critical, not the free radical scavenging property of the compounds [36]. A recent study with a transgenic mouse model has further clarified that* in vivo* BH4 deficiency causes higher IR-induced aortal peroxynitrite formation in the lung tissue sample, as compared to their age-matched wild-type littermates [35]. Hanaue* et al.*, (2007) found IR-induced salivary gland dysfunction in mice is a result of increased formation of peroxynitrite [40]. A significantly higher plasma peroxynitrite level was observed in medical staff exposed to an occupational source of IR [41]. All these data suggesting IR promotes peroxynitrite formation, thereby impairing normal physiologic functions, which might be a result of low BH4 availability after radiation exposure. In addition to IR, BH4 biosynthesis can also be modulated by amino acids, hormones, cytokines, therapeutic agents, substances derived from endothelial cells, oxidants and antioxidants, primarily through modulating the expression of the first and the rate limiting enzyme involved in *de novo* BH4 biosynthetic pathway, called guanosine triphosphate cyclohydrolase-1 (GTPCH1) [42,43,44,45,46,47,48].

## 4. Radiation Effect on GFRP and the Molecular Signaling Pathways that Modulates BH4 Biosynthesis

The activity of GTPCH1 has been shown to be inhibited by GTPCH1 Feedback Regulatory Protein (GFRP) by a protein-protein interaction in presence of the end-product in the *de novo* BH4 biosynthetic pathway, BH4, thereby forming a negative feedback loop [48]. The expression of GFRP can be expected to inversely modulate BH4 biosynthesis. Indeed, a number of previously published articles have revealed that pro-inflammatory stimuli- or H_2_O_2_-mediated GFRP suppression promotes BH4 bioavailability in endothelial cells [49,50]. However, the effect of IR on GFRP is not well documented in literature. Recent studies have shown GFRP expression was increased at the mRNA level in lung and liver tissue samples of wild-type mice after 8.5 Gy of TBI, indicating a GFRP-mediated inhibition of GTPCH1 activity as a possible mechanism of BH4 suppression after IR exposure [35,51]. Moreover, our group previously demonstrated vitamin E analog gamma tocotrienol (GT3) prevented the reduction of lung BH4 bioavailability on day 3.5, followed by a compensatory increase in BH4 level on day 14 and 21 after 8.5 Gy of TBI [36]. The GT3-mediated attenuation of decreased BH4 bioavailability after irradiation could be attributed to GFRP differential regulation. Our* in vitro* data clearly indicate that GT3 treatment significantly reduces GFRP expression at the mRNA and protein level, as well as, significantly prevented GFRP-GTPCH1 binding in endothelial cells, without modulating the expression of GTPCH1 [36]. However, it has been shown BH4 synthesis is modulated in a GFRP independent manner. Tatham* et al.*, (2009) reported that cellular GFRP expression is not a determining factor for BH4 availability; rather GTPCH1 expression is the primary regulator [52]. Shiraishi* et al.*, (2011) also showed that a type-III phosphodiesterase inhibitor suppressed cytokine-induced BH4 biosynthesis in endothelial cells by inhibiting GTPCH1 activity without modulating GFRP expression [53]. The mechanism by which IR regulates BH4 biosynthesis via modulating other molecular signaling pathways is not well characterized.

IR is known to activate the NF-κB pathway in different cell types depending on the radiation dose [54]. Activated NF-κB translocates into nucleus, binds to a specific site on DNA and regulates the expression of various genes, including the inducible form of NOS enzyme (iNOS), which is a major source of NO production under oxidative stress condition. Hanaue* et al.*, (2007) has shown that IR-induced oxidative stress up-regulates iNOS-mediated NO production in salivary gland cells and is the major cause of salivary gland dysfunction [40]. Although IR is known to stimulate the activity of both iNOS and eNOS, the mechanism of enhanced activity is completely different. Radiation-induced eNOS activation is regulated by ATM and HSP-90 [55], while it has been recently shown that iNOS activation via NF-κB pathway occurs immediately following radiation exposure (within 2 h) [56]. IR promotes iNOS synthesis in a NF-κB-dependent manner inducing NO production, which may in turn interact with IR-induced superoxide to form peroxynitrite [57,58]. Peroxynitrite has the potential to oxidize BH4, suggesting a critical role of the NF-κB pathway in modulating BH4 bioavailability after IR exposure [59]. Furthermore, Huang* et al.*, (2005) reported the NF-κB pathway, in combination with the STAT signaling pathway, plays a critical role in regulating GTPCH1 expression [60]. JAK-STAT pathway activation has been shown to be a result of IR-induced DNA damage, which indicates a possible involvement of JAK-STAT pathway in IR-induced BH4 deficiency [61].

## 5. Possible Mechanisms of IR-Induced BH4 Deficiency-Mediated Fibrosis Development

**Figure 1:**
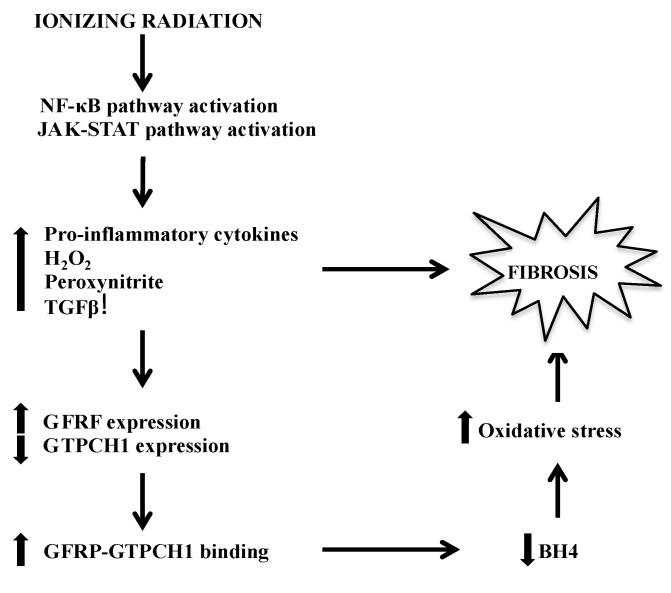
Major pathways by which BH4 deficiency-mediated fibrosis manifests following IR. BH4, tetrahydrobiopterin; IR, Ionizing radiation

Various tissues are known to develop a fibrotic disorder months after the initial radiation insult, which is a consequence of delayed radiation effects. Chronic oxidative stress and inflammation are known to help drive the late effects and fibrosis, suggesting that IR-induced BH4 deficiency might play a direct and/or indirect role in tissue fibrosis (Figure 1).

The molecular mechanism of liver injury as a consequence of IR exposure, especially in the context of concomitant chemotherapy, is not well understood [62,63,64,65]. IR-induced BH4 deficiency promotes formation of peroxynitrite, which is known to play a major role in tissue fibrosis [66], consequently it could directly increase fibrotic response of this tissue following radiation. Furthermore, it has been demonstrated that liver fibrosis from late radiation effects correlated with the increase in intracellular TGFβ1 levels [67]. TGFβ1 activation and overexpression following chronic inflammation has been shown to increase significantly in the IR exposed livers. Anscher* et al.*, also showed TGFβ1 injections in irritated livers produced a significant fibrotic reaction. Suppression of TGFβ1 activation prevents IR-induced fibrosis in mice [58]. Because peroxynitrite also augments the release of matrix metalloproteinase, a critical factor for fibrotic development via activation of TGFβ1 and NF-κB pathway, it is logical to hypothesize that decreased levels of BH4 following IR exposure might indirectly contribute to liver fibrosis following radiation as well.

In addition to liver fibrosis, TGFβ1 serum levels were recently examined to determine the extent of fibrosis in breast cancer patients following radiation. The levels correlated with the severity of fibrosis observed and may be used as a biomarker [68]. Deficient NO production is one of the critical indicators of fibrotic diseases in tissues such as heart, which may be inadequate after radiation exposure. Patients with fibrotic disorder have been shown to produce low NO [69,70]. A study utilizing an animal model has shown BH4 supplementation attenuates fibrosis development [71]. Therefore, BH4 oxidation after radiation exposure might expect to attenuate NO production via “eNOS uncoupling” and subsequently help in disease progression in various tissues [59,72,73,74].

## 6. Metabolomics Enhances Our View of BH4’s Role in Radiation Response

Metabolomics enables us to determine the precise changes in the metabolites endogenous levels following exposure to different doses and quality of radiation over time. We have recently used a metabolomics approach to characterize metabolic changes in an otherwise asymptomatic liver tissue following gamma radiation exposure [51]. We applied a combination of untargeted and targeted quantitative mass spectrometry to study the changes in IR-induced liver injury in mice overexpressing Gfrp, which resulted in decreased levels of BH4 as we previously discussed in this review. Our data demonstrated that when exposed to a non-lethal dose of IR, Gfrp overexpressing mice exhibited a significant accumulation of metabolites associated with oxidative stress and lipid peroxidation. Moreover, IR exposure exacerbated dysregulation of lipid metabolism and caused increased expression of genes that facilitate liver fibrosis in a time dependent manner [51].

All the markers indicative of liver damage and fibrosis were significantly higher in IR exposed Gfrp knock-in mice compared to their irradiated wild type littermates. In addition, using metabolomics we were able to detect these biomarkers at very early time points. Gfrp transgenic mice demonstrated decreased levels of glutathione and bilirubin, but increased levels of glycoholic acid, *N*-arachidonyl taurine, FAD and bile acids as early as 24 to 96 h. The significance of these findings is two-fold. First, our data strongly exhibited the impact of BH4 bioavailability in regulating redox homeostasis and lipid/fatty acid metabolism against IR-induced stress. Secondly, we effectively used metabolomics to show significant changes of metabolites in liver, a late responding tissue, quickly following IR exposure [51].

Others also used similar approaches to define changes in metabolites in other tissues. The response of skin tissue to low-dose radiation mimics the radiobiology of tissues exposed to high-dose radiation, suggesting that biomarkers may be available even at low-dose radiation exposures [75]. Metabolomic profiling of human skin tissue cultures exposed to low dose (< or equal to 10 cGy) ionizing radiation revealed dose and time dependent perturbations in pathways of DNA/RNA damage and repair and lipid and energy metabolism. Changes in metabolites were significantly different from controls at 48 h but not at earlier time points, reflecting a delayed radiation response [75].

Determining changes in biochemical mechanisms and oxidative/reductive metabolism following radiation and how these processes can be manipulated, holds great promise in the radiation field. Identifying novel strategies can enhance therapeutic responses to radiation and mitigate its deleterious effects as well as gather mechanistic information critical for radiation risk assessment for various tissues. Possible biomarkers from selected recent radiation studies using animal models are summarized in Table 1 [19,51,76,77,78,79,80,81].

## 7. Conclusions

Metabolic oxidative stress has been widely accepted as a significant contributor for the early and late effects of IR injury. There is mounting evidence showing how subtle changes in IR induced oxidative/reductive pathways could result in a snowball effect and damage organs unless they are mitigated in a timely manner. Identification of radiation induced asymptomatic tissue injury may lead to a better understanding of metabolic pathway perturbations to be used for the development of mitigators or protectors of radiation induced damage. Developments of new technologies in “omics” fields appear to be very effective in achieving this goal. Metabolomics approaches can be informative not only for developing predictive biomarkers with potential utility to screen at-risk population but also for delineating specific pathway perturbations that would augment the development of effective radio protectors as well as mitigators. Moreover, a systems biology approach which interrogates different levels of cellular expression leading to integration of transcriptomics, proteomics and metabolomics changes offers to provide novel insights and add value towards identification and characterization of biomarkers of IR stress.

**Table 1:** Possible biomarkers reported in selected recent metabolomics studies were either downregulated or upregulated in irradiated animals.

Possible Biomarkers	Method Used	Animal Models	Specimen	Year Reference
**Downregulated in WT mice**	UPLC-ESI-QTOFMS, SID-MRM-MS	Mice	Liver tissue	2014 [51]
Bilirubin, AMD, cytidine, glycerophospholipids, 6-acetyl-d-glucose, C16 sphingomyelin				
**Upregulated in WT mice**				
Secondary bile acids, PS (22:1/0:0)				
**Downregulated in Gfrp mice**				
Glutathione, carnitines, lysoPCs				
**Upregulated in Gfrp transgenic mice**				
Glycocholic acid, *N*-arachidonoyl taurine, flavin adenine dinucleotide, bile acids				
**Upregulated**	GC-TOFMS	Rats	Serum	2012 [76]
Inositol, serine, lysine, glycine, threonine, glycerol				
**Downregulated**				
Isocitrate, gluconic acid, stearic acid				
**Upregulated**	UPLC-ESI-QTOFMS	Rhesus monkeys	Urine	2012 [77]
*N*-acetyltaurine, isethionic acid, taurine, xanthine, hypoxanthine, uric acid, creatine, creatinine, tyrosol sulfate, 3-hydroxytyrosol sulfate, tyramine sulfate, *N*-acetylserotonin sulfate, adipic acid				
**Upregulated**	^1^H NMR	Mice	Serum	2011 [78]
Lactate, amino acids, choline, lipid signals				
**Downregulated**				
Glucose signals				
**Upregulated**	UPLC-ESI-QTOFMS	Rats	Urine	2011 [79]
thymidine, 2′-deoxyuridine, 2′-deoxyxanthosine, *N*^1^-acetylspermidine, *N*-acetylglucosamine/galactosamine-6-sulfate, *N*-acetyltaurine, *N*-hexanoylglycine, taurine, isethionic acid (tentative)				
**Upregulated**	GC-MS	Rats	Urine	2009 [80]
Glyoxylate, threonate, thymine, uracil, *p*-cresol				
**Downregulated**				
Citrate, 2-oxoglutarate, adipate, pimelate, suberate, azelaate				
**Upregulated**	UPLC-QTOFMS	Mice	Urine	2009 [81]
thymidine, 2′-deoxyuridine, 2′-deoxyxanthosine, xanthine, xanthosine				
**Upregulated**	UPLC–TOFMS	Mice	Urine	2008 [19]
*N*-hexanoylglycine, β-thymidine, 3-hydroxy-2-methylbenzoic acid 3-*O*-sulfate, taurine				
